# Navigating appendicitis care during the Covid-19 pandemic: a retrospective cohort study in China

**DOI:** 10.1186/s12893-024-02466-4

**Published:** 2024-05-28

**Authors:** Guang Fu, Zishun Xu, Shao Zhang

**Affiliations:** The First Affiliated Hospital, Department of Gastrointestinal Surgery Hengyang Medical School, University of South China, Hengyang, 421001 China

**Keywords:** COVID-19, Pandemic, Appendicitis, Appendectomy, Delayed surgery

## Abstract

**Background:**

The emergence of the COVID-19 pandemic in December 2019 initiated a global transformation in healthcare practices, particularly with respect to hospital management. PCR testing mandates for medical treatment seekers were introduced to mitigate virus transmission.

**Aims:**

This study examines the impact of these changes on the management of patients with appendicitis.

**Methods:**

We conducted a retrospective analysis of medical records for 748 patients diagnosed with appendicitis who underwent surgery at a tertiary care hospital during two distinct periods, the pre-pandemic year 2019 and the post-pandemic year 2021. Patient demographics, clinical characteristics, laboratory data, surgical outcomes, and hospital stay duration were assessed.

**Results:**

While no significant differences were observed in the general characteristics of patients between the two groups, the time from hospital visit to operation increased significantly during the pandemic. Unexpectedly, delayed surgical intervention was associated with shorter hospital stays but did not directly impact complication rates. There was no discernible variation in the type of surgery or surgical timing based on symptom onset. The pandemic also prompted an increase in appendicitis cases, potentially related to coronavirus protein expression within the appendix.

**Conclusions:**

The COVID-19 pandemic has reshaped the landscape of appendicitis management. This study underscores the complex interplay of factors, including changes in hospital protocols, patient concerns, and surgical timing. Further research is needed to explore the potential link between COVID-19 and appendicitis. These insights are valuable for informing healthcare practices during and beyond the pandemic.

## Introduction

The novel coronavirus SARS-CoV-2 (causing COVID-19) was first reported in China in December 2019, which marked a significant turning point in global health [1]. . By March 2020, the virus had escalated into a pandemic, prompting an urgent need for public health measures to curb its spread. These measures, including social distancing, limited physical contact, and various degrees of confinement, have had profound implications for healthcare delivery. The pandemic’s impact on healthcare was particularly evident in the realm of surgical services. Elective procedures were often deferred to conserve resources and minimize the risk of viral transmission, potentially leading to delays in necessary treatments [2, 3]. The management of surgical emergencies was also disrupted, with patients tending to present with more advanced disease stages due to delayed medical care, thus complicating surgical interventions [4].

Appendicitis is a common surgical emergency, which has traditionally been managed through prompt surgical intervention [5, 6]. However, the pandemic necessitated a reevaluation of this approach as healthcare systems faced unprecedented challenges in resource allocation. Initial reports from the height of the pandemic indicated a decrease in appendicitis cases, possibly due to reduced movement, limited social interactions, and fears of hospital exposure. Additionally, some studies observed a noted shift in the clinical presentation of appendicitis, with more advanced and complicated cases at hospital admission [7–9], while other studies indicated that delay in medical consultation did not necessarily translate into worse outcomes [10, 11]. As we move beyond the pandemic, understanding the impact of lockdown measures on appendicitis is crucial for healthcare planning and preparedness.

This study delves into examining the shift of clinical characteristics and outcomes of appendicitis cases managed by appendectomy before and after pandemic. Our analysis indicated that the pandemic did not increase the rate of complicated appendicitis, but indeed increase delay time from admission to operation. However, this delay did not translated into worse outcomes. By analyzing the data from a tertiary care hospital in China, we seek to provide insights into how healthcare preparedness and emergency response strategies have adapted to unforeseen disruptions.

## Materials and methods

### Study design

We conducted a retrospective analysis of medical records for patients diagnosed with appendicitis (AA) who underwent surgical treatment at the Gastrointestinal Surgery Department of The First Affiliated Hospital of the University of South China. The study period covered the pre-pandemic year 2019 and the post-pandemic year 2021.

### Patient selection

Data of 937 patients who were clinically diagnosed suspected acute appendicitis and underwent operation were retrieved. Including criteria: age over 18, no other severe chronic comorbidities(including hypertension, diabetes, chronic heart disease, chronic liver disease, chronic kidney disease and other chronic conditions that might alter the treatment modality of appendices) and intraoperative diagnosed appendicitis without other surgical emergencies. Excluding criteria: abnormal appendix anatomy, periappendiceal abscess, postoperative pathological diagnosis of benign or malignant appendiceal tumors and those with incomplete data were excluded from the analysis. According to our including and excluding criteria, eventually a total of 748 patients were included in the study.

### Data collection

We collected comprehensive data on the demographic characteristics, medical records, pathological results, surgical data, laboratory test results, and imaging findings of the 748 patients. All data were obtained from the medical records maintained by the hospital.

### Statistical analysis

Prior to statistical analysis, a normality test was performed for the data. For normally distributed data, we reported the mean ± standard deviation analyzed using Student’s *t*-test, while non-normally distributed data was presented using the median and interquartile range and analyzed using Mann-Whitney U test. Categorical variables were assessed for normality and compared using chi-square tests and Fisher’s exact tests.

### Ethical considerations

This retrospective analysis was conducted in accordance with the ethical principles outlined in the Declaration of Helsinki. The study protocol was approved by the institutional review board of The First Affiliated Hospital of the University of South China.

### Statistical software

We performed all statistical analyses using SPSS 26 software.

## Results

A total of 748 patients diagnosed with appendicitis (AA) who underwent surgery were included in this retrospective study. Among them, 326 patients belonged to the pre-pandemic group(2019), while 422 patients were in the post-pandemic group (2021).General characteristics, including age, sex, and associated symptoms, did not exhibit statistically significant differences between the pre-pandemic group and post-pandemic group (Table 1).

**Table 1:** Baseline characteristics of patients underwent appendectomy between pre-pandemic group and pandemic group

					Year					
	2019 (pre-pandemic group)*n* = 326					2021(pandemic group)*n* = 422				
	Median	P25	P75	N(%)		Median	P25	P75	N(%)	**p**
Sex										
Male				166(51%)					219(52%)	0.686
Female				160(49%)					253(48%)	
Age	49.0	32.5	61.0			47.5	34.0	59.0		0.927
Associated symptoms										
Fever				186(57%)					203(52%)	0.130
Nausea				205(63%)					274(65%)	0.512
Vomiting				140(43%)					160(38%)	0.122
Diarrhea and constipation				49(15%)					72(17%)	0.444
Respiratory symptoms				10(3.2%)					16(3.8%)	0.651
White blood cell count (109 /L)	11.1	7.5	14.6			10.9	7.5	15.4		0.804
Neutrophil index(%)	83.7	72.8	89.2			79.5	67.7	87.5		0.144
C-reactive protein (mg/dL)	34.1	6.2	83.8			38.4	10.1	93.6		0.766
Alanine aminotransferase(U/L)	12.9	8.6	18.5			11.7	9.1	16.6		0.966
Glutamate aminotransferase(U/L)	17.6	14.1	22.6			17.6	14.5	23.0		0.356
Time from symptom onset to hospital visit (days)	1.1	1.0	3.0			1.0	0.6	3.0		0.584

There were no statistically significant differences between the pre-pandemic group and post-pandemic group in terms of the time of time from symptom onset to hospital visit and laboratory test results on arrival (Table 1). In terms of imaging findings, we observed no difference between the pre-pandemic group and post-pandemic group regarding the presence rate of appendiceal fecal stones and appendix diameter (Table 2).

**Table 2:** Imaging data of of patients in pre-pandemic group and pandemic group

					Year					
	2019 (pre-pandemic group)*n* = 326					2021(pandemic group)*n* = 422				
	Median	P25	P75	N(%)		Median	P25	P75	N(%)	**p**
Diagnostic methods										
Computed Tomography				183(56%)					224(53%)	0.715
Ultrasonic imaging				46(14%)					25(6%)	0.075
Both of the above				98(30%)					215(51%)	0.141
Appendix diameter(mm)	9.6	7.9	11.5			9.1	6.3	11.9		0.366
Appendiceal fecal stones				108(33%)					173(41%)	0.260

However, several key findings emerged during the post-pandemic period. The time from hospital visit to operation significantly increased in the post-pandemic group (*p* = 0.015) (Table 3). Notably, patients with chronic appendicitis underwent operation increased during the post-pandemic period (*p* = 0.009) (Table 3).Interestingly, no significant differences were observed in the type of operation, time of operation, placement of abdominal drainage tube, and postoperative complication rates between the two groups (Table 4). However, significant shorter length of hospital stay in post-pandemic group was observed compared to the pre-pandemic group (*p* = 0.003) (Table 4).

**Table 3:** Intraoperative data of of patients in pre-pandemic group and pandemic group

					Year					
	2019 (pre-pandemic group)*n* = 326					2021(pandemic group)*n* = 422				
	Median	P25	P75	N(%)		Median	P25	P75	N(%)	**p**
Time from hospital visit to operation (hours)	6.5	4.5	9.4			7.6	6.3	8.9		0.015
Intraoperative diagnosis										
Simple appendicitis				49(15%)					80(19%)	0.116
Purulent appendicitis				215(66%)					249(59%)	0.031
Perforated appendicitis				29(9%)					25(6%)	0.093
Chronic appendicitis				33(10%)					68(16%)	0.009
Type of surgery										
Laparoscopy appendicectomy				319(98%)					418(99%)	0.498
Laparotomy appendicectomy				8(2.5%)					6(1.5%)	
Whether to have an indwelling abdominal drain										
Yes				199(61%)					215(51%)	0.157
No				127(39%)					207(49%)	

**Table 4:** Postoperative data of of patients in pre-pandemic group and pandemic group

					Year					
	2019 (pre-pandemic group)*n* = 326					2021(pandemic group)*n* = 422				
	Median	P25	P75	N(%)		Median	P25	P75	N(%)	**p**
Postoperative complications				4(1.2%)					9(2.2%)	0.550
haemorrhage				0					0	
Wound infection				4(1.2%)					8(1.8%)	
ileus				0					1(0.2%)	
appendix stump inflammation				0					0	
Intestinal fistula				0					0	
Duration of surgery (min)	45.0	30.0	60.0			40.0	30.5	55.0		0.304
Length of stay (days)	4.7	3.7	6.0			3.9	2.9	4.8		0.003

**Fig. 1:**
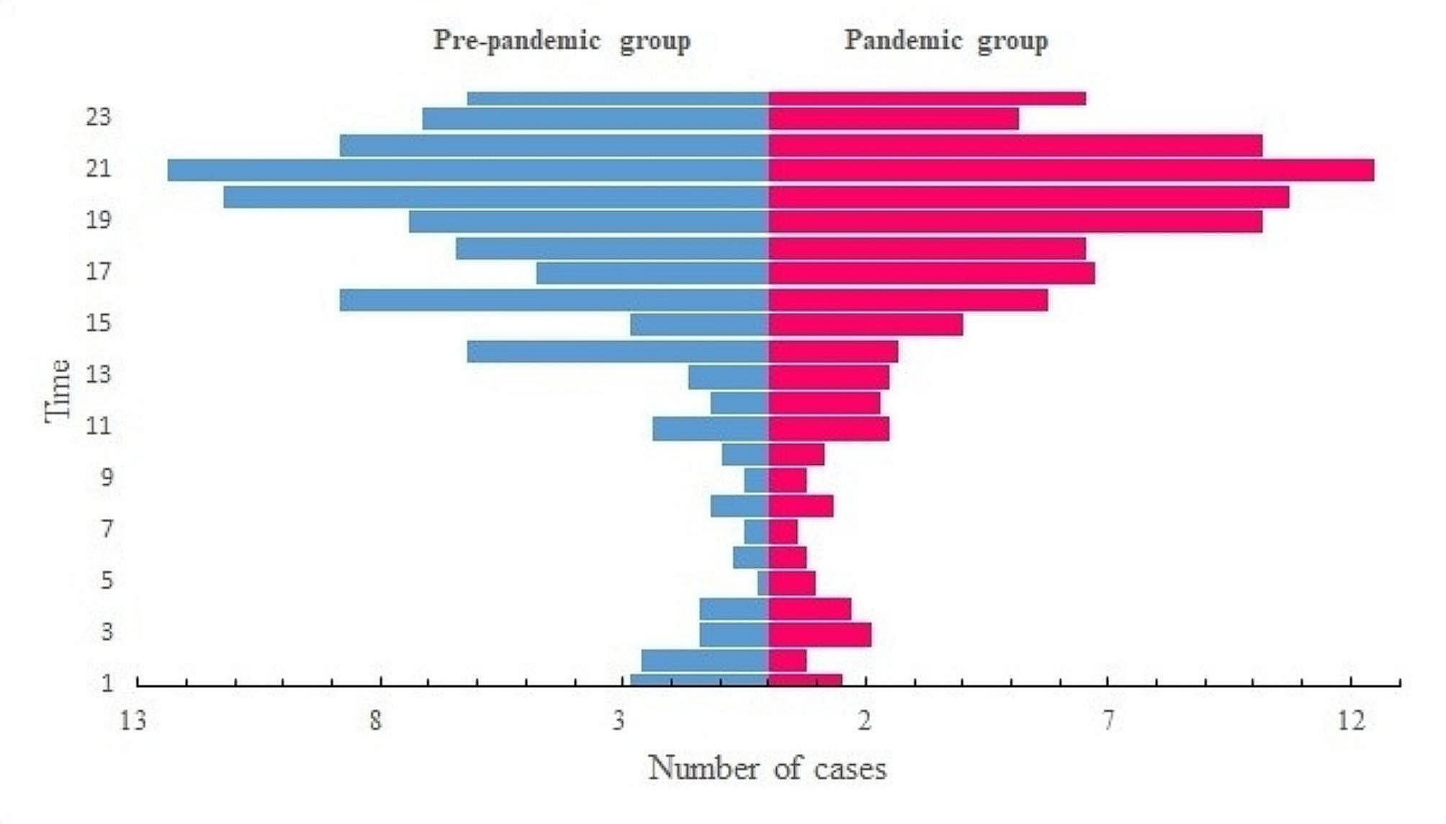
The distribution of operation time during 24 h between pre-pandemic group and pandemic group. Most of appendectomy were performed during the night time and the pandemic did not impact this pattern

We were also interested in the question whether pandemic would change the timing of appendectomy during the day and night. Our data revealed that the majority of surgical procedures in both the pre-pandemic and pandemic periods were performed during the nighttime hours and the pandemic did not shift the pattern(Fig. 1).

## Discussion

Appendicitis (AA) can be classified as simple or complex, the latter encompassing perforation and suppuration, along with acute episodes of chronic appendicitis [5]. The traditional management approach involves appendectomy, often conducted laparoscopically, with reported successful cure rates of 73.4% for conservative treatment and 97.4% for surgical intervention [12]. However, the exigencies of the pandemic have led to a re-evaluation, with an observed shift towards the utilization of antibiotics as the primary treatment in certain regions [13]. This approach is intended to reduce the duration of hospital stays and lower the risks of viral transmission, despite the potential for subsequent re-hospitalization and additional surgical interventions [14, 15]. .

The pandemic has instigated a significant adaptation in the global healthcare system, particularly in hospital management practices. The introduction of mandatory PCR testing for medical treatment seekers, except in emergency cases, has notably influenced the management of appendicitis, a common cause of abdominal pain and a leading indicator for emergency abdominal surgery worldwide [16]. While studies in some regions reported increased time from symptom onset to hospital visit and more complex cases due to these measures [7, 17], our study, in alignment with other Chinese retrospective cohorts, found no significant differences in the management of appendicitis in China [10]. These findings suggest minimal influence on appendicitis patients’ seeking behaviors in China, potentially attributed to cultural nuances.

A critical finding of our study is the prolonged interval from emergency room admission to surgery during the pandemic, primarily due to the implementation of additional procedures such as real-time PCR testing [18], which takes approximately 4–6 h [10]. Although delayed surgical intervention can potentially exacerbate inflammation and complications, our study observed no direct impact on complication rates. Similar results were also noted in recent multicenter trials [19, 20] and meta-analyses [21] indicating that delayed surgery, even up to 24 h, did not increase post-operative complications.

Our data revealed that the majority of appendectomies were scheduled during nighttime hours, with no discernible shift in this pattern due to the pandemic. Prior research has indicated similar complication rates for appendectomies performed during daytime versus nighttime [22–24]. Given the new findings that delayed surgery does not significantly impact postoperative complications, this raises questions about the necessity of scheduling appendectomies late into the night. The practice of postponing appendectomies until daytime could potentially allow surgeons to rest during night shifts, thereby enhancing their daytime performance, although this perspective warrants further examination.

Interestingly, our study observed an uptick in appendicitis cases in the post-pandemic year 2021 compared to the pre-pandemic year 2019, which contrasts with reports from Western countries. This discrepancy may be partially ascribed to differences in healthcare systems between China and the West. While Western patients tended to seek care locally during the pandemic, our tertiary hospital experienced an influx of referrals, possibly contributing to the increase in appendicitis cases. Additionally, there is emerging evidence suggesting a potential link between coronavirus infection and the development of appendicitis, possibly through the presence of ACE2 receptors in the appendix mucosal cells [25]. However, a study published in 2022 conducted virologic analysis of appendectomy samples from children and found no SARS-CoV-2 in appendicitis tissues with pandemic’s backdrop [26]. Whether SARS-CoV-2 is a frequent etiologic factor in appendicitis warrants further investigation [27].

Notably, despite the surge in appendicitis cases post-pandemic, we observed significantly shorter hospital stays, which may be linked to patients’ heightened awareness and concerns about infection risks during the pandemic.

The study’s limitations should be acknowledged. As a single-center study, inherent selection biases may limit the generalizability of the findings. Variations in antibiotic selection and surgical strategies among different surgeons could introduce confounding factors. Furthermore, patients’ healthcare-seeking behaviors may evolve over time with policy adjustments, and our one-year data collection may not fully capture the complete impact of COVID-19 on the treatment of appendicitis.

Our study sheds light on the multifaceted impact of the COVID-19 pandemic on the management of appendicitis, highlighting the complex interplay of hospital protocols, patient concerns, and surgical timing. Further research is warranted to explore the potential link between COVID-19 and appendicitis, informing improved healthcare practices during and beyond the pandemic.

## Data Availability

The dataset used in this study is available from the corresponding author upon appropriate request.

## References

[1] Wu Z, McGoogan JM (2020). Characteristics of and important lessons from the Coronavirus Disease 2019 (COVID-19) outbreak in China: Summary of a report of 72 314 cases from the Chinese Center for Disease Control and Prevention. JAMA.

[2] Tebala GD (2022). Emergency surgery admissions and the COVID-19 pandemic: did the first wave really change our practice? Results of an ACOI/WSES international retrospective cohort audit on 6263 patients. World J Emerg Surg.

[3] Leite C (2020). Gastrointestinal emergency care during the COVID-19 pandemic: rapid communication. Rev Assoc Med Bras (1992).

[4] Bickell NA (2006). How time affects the risk of rupture in appendicitis. J Am Coll Surg.

[5] Di Saverio S (2020). Diagnosis and treatment of acute appendicitis: 2020 update of the WSES Jerusalem guidelines. World J Emerg Surg.

[6] Ruffolo C (2013). Acute appendicitis: what is the gold standard of treatment?. World J Gastroenterol.

[7] Grossi U (2022). Changes in hospital admissions and complications of acute appendicitis during the COVID-19 pandemic: a systematic review and meta-analysis. Health Sci Rev (Oxf).

[8] Kumaira Fonseca M (2020). Impact of COVID-19 outbreak on the emergency presentation of Acute Appendicitis. Am Surg.

[9] Rosenthal MG (2021). Where did all the appendicitis go? Impact of the COVID-19 pandemic on volume, management, and outcomes of Acute Appendicitis in a Nationwide, Multicenter Analysis. Ann Surg Open.

[10] Wang WD (2022). Analysis of appendicitis management during COVID-19 pandemic: a study of Chinese adult cohorts. Front Surg.

[11] Zhang P, Zhang Q, Zhao HW (2022). COVID-19 pandemic changed the management and outcomes of acute appendicitis in northern Beijing: a single-center study. World J Clin Cases.

[12] Wilms IM et al. Appendectomy versus antibiotic treatment for acute appendicitis. Cochrane Database Syst Rev, 2011(11): CD008359. 10.1002/14651858.CD008359.pub2 22071846

[13] Collard M (2020). Antibiotics alone as an alternative to appendectomy for uncomplicated acute appendicitis in adults: changes in treatment modalities related to the COVID-19 health crisis. J Visc Surg.

[14] Salminen P (2018). Five-year follow-up of antibiotic therapy for uncomplicated Acute appendicitis in the APPAC Randomized Clinical Trial. JAMA.

[15] Habib Bedwani N (2023). Two-year outcomes of conservatively managed appendicitis during the COVID-19 pandemic-a multicentre cohort study. Langenbecks Arch Surg.

[16] Stewart B (2014). Global disease burden of conditions requiring emergency surgery. Br J Surg.

[17] Burgard M (2021). An effect of the COVID-19 pandemic: significantly more complicated appendicitis due to delayed presentation of patients!. PLoS ONE.

[18] Thomas MB (2023). Prioritizing rapid COVID-19 testing in emergency general surgery patients decreases burden of inpatient hospital admission. Trauma Surg Acute Care Open.

[19] Patel SV et al. Delayed vs. early laparoscopic appendectomy (DELAY) for adult patients with Acute appendicitis: a randomized controlled trial. Ann Surg, 2023. 10.1097/SLA.0000000000005996 37436871

[20] Jalava K (2023). Role of preoperative in-hospital delay on appendiceal perforation while awaiting appendicectomy (PERFECT): a nordic, pragmatic, open-label, multicentre, non-inferiority, randomised controlled trial. Lancet.

[21] Li J (2019). Effect of Delay to Operation on outcomes in patients with Acute Appendicitis: a systematic review and Meta-analysis. J Gastrointest Surg.

[22] Alore EA (2018). Population-level outcomes of early versus delayed appendectomy for acute appendicitis using the American College of Surgeons National Surgical Quality Improvement Program. J Surg Res.

[23] Monttinen T (2021). Nighttime Appendectomy is safe and has similar outcomes as Daytime Appendectomy: a study of 1198 appendectomies. Scand J Surg.

[24] Shah AA (2022). Daytime versus nighttime laparoscopic appendectomy in term of complications and clinical outcomes: a retrospective study of 1001 appendectomies. Heliyon.

[25] Demyashkin G (2022). Angiotensin-converting enzyme 2 and furin expression in the appendix of children with COVID-19. Surg Infect (Larchmt).

[26] Jiang Y (2022). SARS-CoV-2 infection is not Associated with Pediatric Appendicitis. Pediatr Infect Dis J.

[27] Puoti MG (2021). SARS-CoV-2 and the gastrointestinal tract in children. Front Pediatr.

